# The relationship between optic nerve head deformation and visual field defects in myopic eyes with primary open-angle glaucoma

**DOI:** 10.1371/journal.pone.0209755

**Published:** 2018-12-31

**Authors:** Chih-Heng Hung, Shwu-Huey Lee, Szu-Yuan Lin, Shun-Ling Lin, Yi-Chun Chen

**Affiliations:** Department of Ophthalmology, Cathay General Hospital, Taipei, Taiwan; University of California San Diego, UNITED STATES

## Abstract

**Purpose:**

To investigate the relationship between the morphologic features of myopic optic nerve head (ONH) and visual field (VF) defects in myopic subjects with primary open-angle glaucoma (POAG) by intraindividual comparison.

**Methods:**

Myopic POAG subjects with unilateral glaucomatous VF defect were recruited. The morphologic features of myopic ONH, including optic disc tilt, optic disc rotation, and β-zone parapapillary atrophy (PPA) were measured from color fundus photographs. The comparisons were performed between the eyes with VF defects and the contralateral eyes without VF defects. Logistic regression analysis was performed to investigate the relationship between various ocular parameters and the presence of VF defects.

**Results:**

We retrospectively included 100 eyes of 50 myopic POAG subjects. (Mean age: 50.1 ± 10.0 years). The tilt ratio was similar between the paired eyes. The degree of optic disc rotation (12.96 ± 7.21°) in eyes with VF defects were statistically greater than the contralateral eyes (6.86 ± 4.30°; *P* < 0.001) without VF defect. The β-zone PPA-to-disc area ratio was significantly greater in eyes with VF defects than the contralateral eyes (*P =* 0.024) without VF defect. In multivariate logistic regression analysis, the greater degree of optic disc rotation was significantly associated with the presence of VF defects (*P* < 0.001). However, tilt ratio, β-zone PPA-to-disc area ratio, refractive error, and axial length were not associated with the presence of VF defects.

**Conclusions:**

Among the morphologic features of myopic ONH, only the greater degree of the optic disc rotation was associated with the presence of VF defects in myopic subjects with POAG.

## Introduction

Myopia is on the rise around the world, especially in East Asian and Southeast Asian countries. [1] In Taiwan, five nationwide myopia surveys covering school children aged between 6 and 18 demonstrated the prevalence of myopia was more than 80% by the age of 18 years. The prevalence of high myopia (spherical equivalence [SE] < -6 diopters [D]) was 21% in 18 years old students. [2]

In multiethnic populations, myopia has been identified as a risk factor for primary open-angle glaucoma (POAG) and the reasons may be due to the weakened structural changes associated with deformation of the optic nerve head (ONH) in myopia. [3–11] A systemic review and meta-analysis of 13 studies involving 48,161 individuals reported that the pooled odds ratios (ORs) of association between myopia and glaucoma were 1.88 (95% confidence interval [CI], 1.60–2.20) for all myopia. [4] In the Beijing eye study, the prevalence of glaucomatous optic nerve change in eyes with high myopia (refractive error < -6D) was significantly (OR, 3.5; 95% CI, 1.71–7.25) higher than in eyes with low myopia (refractive error ≥ -3D), and it was significantly (OR, 7.56; 95% CI, 3.98–14.35) higher than eyes with emmetropia. [8]

On the other hand, Quigley *et al* did not find a clear relationship between myopia and the development of glaucoma in the 647 ocular hypertensive subjects. [10] Jonas *et al* also showed the myopia did not play a major role for the amount of glaucomatous optic neuropathy in non-highly myopic (SE > -8D) patients with open-angle glaucoma. [11] The possible explanations for the conflict results of the relationship between myopia and glaucoma may due to different study populations and study designs. The other relevant reason could be that, the previous studies used refractive error or axial length (AL) to describe the severity of myopia. As the influence of myopic axial elongation on ONH may be different in each myopic subject, the more myopic eyes are not necessarily associated with more severe ONH deformation. Therefore, the refractive error or AL cannot exactly reflect the severity of structural changes of ONH in myopic eyes. The actual structural changes of ONH associated with myopia may be more relevant to the susceptibility to glaucoma in myopic eyes, rather than the refractive error or AL.

The ONH deformation associated with myopia included larger and more oval-shaped disc, optic disc tilt (particularly temporal disc tilt), optic disc rotation, and larger area of parapapillary atrophy (PPA) by ophthalmoscopical evaluation. [12,13] In Japanese subjects with high myopia (SE < -8D or AL ≥ 26.5mm), Nagoka *et al* suggested the increase of prevalence of POAG was primarily associated with larger optic disc size beyond a disc area of 3.8 mm^2^, not with axial elongation itself. [14] Chen *et al* investigated 375 Taiwanese with POAG and high myopia (SE < -6D). [15] They reported that greater disc tilt was associated with more severe glaucomatous optic neuropathy and disc area had no relationship with VF defects. Kimura *et al* found high myopic and tilted disc was associated with paracentral scotoma in highly myopic eyes with early glaucoma. [16] Sung *et al* also reported that greater disc tilt and lesser disc rotation were associated with the worse parafoveal VF defect in myopic patients with early normal tension glaucoma. [17]

The other important myopic ONH deformation correlated to glaucoma is optic disc rotation. [18–20] In Korean, normal subjects with myopia and greater optic disc rotation was significantly correlated with longer AL. [21] Park *et al* demonstrated inferior optic disc rotation predicted superior visual field (VF) defect and superior optic disc rotation predicted inferior VF defect in myopic subjects with normal-tension glaucoma. [18] They proposed that the superior rotation of optic disc could place stress on the superior nerve axons, resulting in damage to the retinal nerve fiber layer (RNFL) in the superior region, whereas inferior rotation of optic disc could induce RNFL damage in the inferior region.

Lee *et al* investigated young myopic subjects with unilateral glaucomatous VF defects and found the greater degree of optic disc rotation was associated with greater glaucomatous VF defects. [19] Sung *et al* reported optic disc rotation-VF defect correspondence was an important protective factor for VF progression in myopic normal-tension glaucoma with hemifield VF defects. [20] Which means if the eyes with inferior rotation of optic disc had an superior RNFL defect and corresponding inferior VF defect, the VF defect tended to be progressive.

The PPA is associated with a variety of ocular conditions, including aging, myopia, and glaucoma. [22,23] β-zone PPA is spatially correlated with retinal nerve fiber layer defect and VF damage in patients with glaucoma. [24,25] In myopic eyes, the peripapillary crescent, which refers to as hypo-pigmented area with visible sclera cannot be easily differentiated from the β-zone PPA. Thus, the β-zone PPA investigated by ophthalmological evaluation probably included β-zone PPA and/or myopic crescents in myopic eyes. As β-zone PPA is clinically visible this might include β-zone PPA and myopic crescents, the clinical examination of β-zone PPA for glaucoma evaluation could be confused by the coexistence of β-zone PPA and myopic crescents in myopic eyes with glaucoma.

In myopic subjects with POAG, we noted more VF defects were not directly correlated to the eyes with more myopia. Sawada *et al* demonstrated that the eyes with more VF defects were associated with more optic disc tilt and β-zone PPA in myopic subjects with POAG. However, the optic disc rotation and refractive error were not correlated with the severity of VF damage. [26] Thus, the axial elongation of myopia itself might not be the direct impact factor associated with glaucoma but the severity of ONH deformation related to myopia is.

Among the most investigated ONH deformation in myopic eyes, including optic disc tilt, optic disc rotation, and β-zone PPA, which one is more relevant to glaucoma remains controversial. In Taiwan, with very high prevalence of myopia, we face the increasing challenge of diagnosing glaucoma in myopic subjects. Knowing which ONH deformation correlates more to glaucoma can help us diagnose glaucoma in myopic subjects. Therefore, we would like to find out what structural changes of myopic ONH may influence the presence of glaucomatous VF defects. We designed the intra-individually paired eye comparisons in myopic subjects to minimize the systemic confounding factors such as age, gender, ethnicity, heredity, systemic vascular factors, and cerebrospinal fluid pressure. We compared the optic disc tilt, optic disc rotation, and β-zone PPA between the paired eyes to find out which structural changes of ONH contributing to the glaucomatous VF defects in myopic subjects. In addition, we investigated various ocular parameters that may be associated with the presence of VF defects.

## Methods

### Patients

This retrospective cross-sectional study was approved by the Institutional Review Board of the Cathay General Hospital (protocol number CGH-P102044) and was carried out in accordance with the tenets of the Declaration of Helsinki. Informed consent was considered not required by Institutional Review Board and all data were fully de-identified before accessing them. We reviewed the medical records of consecutive patients attending the outpatient clinics of the Department of Ophthalmology, Cathay General Hospital from January 2012 to December 2012 and subjects that met predefined eligibility criteria were included.

All subjects underwent comprehensive ophthalmic examinations including (1) Best-corrected visual acuity (BCVA). (2) Refraction measured by a KR-3000 Auto Refractometer (Topcon, Tokyo, Japan). Three measurements were recorded and the mean value was calculated for statistical analysis. SE is defined as spherical plus half signed cylindrical refractive error. (3) Slit-lamp biomicroscopy. (4) Intraocular pressure (IOP) measured by Air-puff tonometry (Topcon CT-80, Topcon Corporation, Tokyo, Japan). Mean value of the three readings was used for analysis. (5) Gonioscopy. (6) Dilated fundus examination. (7) Color fundus photography obtained with a Canon CR-6 Non-Mydriatic Fundus Camera (Canon, Kanagawa, Japan). (8) Central corneal thickness (CCT) measured by the Orbscan corneal topography system version 3.00 (Orbtek Inc., Salt Lake City, UT, USA). (9) AL measured by an ophthalmic ultrasound biometer (PacScan 300, Lake Success, NY, USA). (10) Automated perimetry measured by Octopus 101 perimeter (Interzeag AG, Schlieren, Switzerland). (11) RNFL thickness and ONH measured by the Cirrus HD spectral-domain optical coherence tomography (SD-OCT) device (version 5.01 Carl Zeiss Meditec, Dublin, CA, USA). Signal strength of 6 or more was considered acceptable. The untreated IOP determined by the average of at least two measurements before treatment was use for analysis.

Automated perimetry was performed using the tendency oriented perimetry (TOP) algorithm. The glaucomatous VF defect was defined as (1) the mean defects (MD) > 2 dB and/or the loss variance (LV) > 6dB; (2) the presence of at least three contiguous non-edge abnormal points with *P* < 5% probability of being normal (allowing the two nasal step edge points) and one of these points with a *P* < 1% on the corrected probability plot within the same hemifield, which was confirmed on two consecutive reliable tests. [27] VF test results with >15% of false-positive responses or false-negative responses or reliability factor were considered unreliable.

The inclusion criteria were as follow: (1) BCVA ≥ 20/30. (2) SE ≤ -0.5 D and AL ≥ 24.0 mm. (3) The paired eyes have asymmetric AL. (4) Open angles on gonioscopy. (5) Glaucomatous optic disc appearance was defined as diffuse or focal neuroretinal rim thinning, diffuse or focal RNFL defects, or inter-eye vertical cup-to-disc ratio asymmetry ≥ 0.2. (6) Corresponding glaucomatous VF defects in one eye.

The exclusion criteria on the basis of any of the following: (1) Previous ocular surgery. (2) A history of ocular trauma. (3) Corneal opacity. (4) Vitreoretinal diseases. (5) Pathologic myopic fundus changes. (6) Other optic nerve disease except for glaucoma. (7) Horizontally oval disc and tilted disc syndrome. (8) Systemic or neurologic diseases that may affect the VF.

### Measurement of optic disc tilt, optic disc rotation, and β-zone parapapillary atrophy area

Optic disc morphologic parameters were measured on color fundus photographs by two authors (CHH, YCC) masked to the VF results using the National Institutes of Health image-analysis software (ImageJ version 1.40; available at http://rsb.info.nih.gov/ij/index.html; developed by Wayne Rasband, National Institutes of Health, Bethesda, MD). The average values of the two examiners were used for statistical analysis.

**Fig 1:**
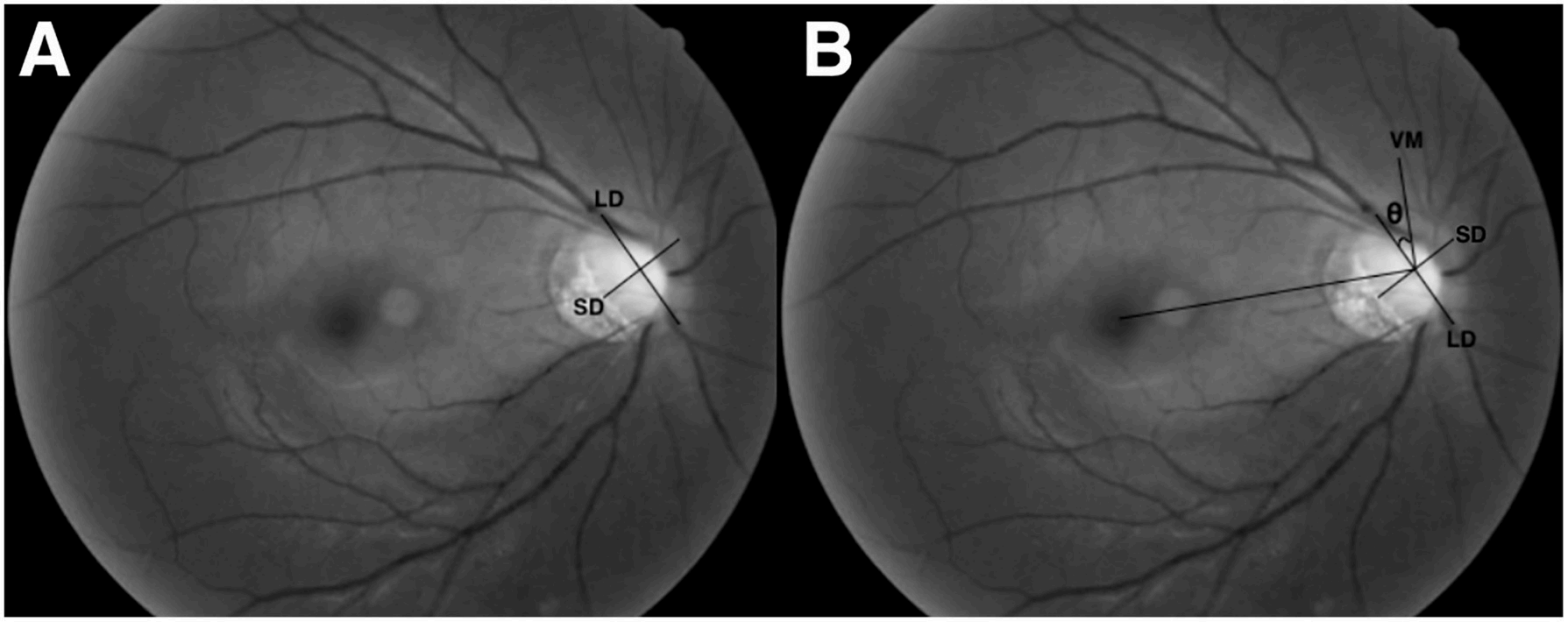
Measurement of tilt ratio and rotation degree. (A) Tilt ratio was defined as the ratio between the longest diameter (LD) and shortest diameter (SD) of the optic disc, and the LD was orthogonal to the SD. (B) Rotation degree (θ) was measured between the LD and the vertical meridian (VM), which was identified as a vertical line (90°) from a reference line connecting the fovea and the center of the optic disc.

Optic disc tilt was determined by the tilt ratio, defined as the ratio between the longest diameter and shortest diameter of the optic disc (Fig 1A). [18] Optic disc rotation was defined as the deviation of the longest diameter of the disc from the vertical meridian, which was a vertical line perpendicular to a reference line connecting the fovea and the center of the disc. The angle between the vertical meridian and the longest diameter of the disc was termed the degree of rotation (Fig 1B). [18] Positive and negative angles indicated the presence of inferotemporal and supranasal rotation, respectively. The absolute values of rotation angle were used for analysis to avoid compensation of the positive and negative values of rotation angle. β-zone PPA was defined as an inner crescent of chorioretinal atrophy with visible sclera and choroidal vessels. β-zone PPA-to-disc area ratio was defined as the ratio between the pixel areas of the β-zone PPA area and the disc area and was used for analysis by minimizing the effect of ocular magnification error.

### Statistical analysis

The demographic and ocular parameters were analyzed by using descriptive statistics (number, percentage of each categorical variable, and the mean ± standard deviation of each continuous variable). The severity of VF defects was evaluated according to the MD of the VF test. The paired eyes were divided into the eyes with VF defects group and the eyes without VF defects group. A paired *t* test was used to assess the differences between the two groups.

Univariate and multivariate logistic regression analyses were performed to investigate various ocular parameters that may be associated with the presence of VF defects. For this analysis, the independent variables were SE, AL, IOP, CCT, tilt ratio, optic disc rotation degree, β-zone PPA to disc area ratio, disc area, and RNFL thickness. The dependent variable was the presence of VF defects. All parameters in the univariate analysis were included in the multivariate logistic regression analysis model.

In addition, all subjects were divided into two subgroups according to the severity of myopia determined by AL and the presence of VF defects. There were 38 subjects (76%) with more myopic eyes accompanying with VF defects and the contralateral less myopic eyes without VF defects classified as subgroup one. The other 12 subjects (24%) with less myopic eyes accompanying with VF defects and the contralateral more myopic eyes without VF defects were classified as subgroup two. In both subgroups, a paired *t* test was used to assess the differences of the various ocular parameters.

To investigate the relationship between the direction of the optic disc rotation and the location of VF defects, the eyes with hemifield VF defects were divided into superior and inferior defects subgroups. The definition of hemifield VF defect included glaucomatous defects in one hemifield and the other intact hemifield consisting of no test points with a probability level less than 2% and no clusters of ≥ 3 adjacent points with a probability of less than 5% in the corrected probability plot. Agreement between the direction of optic disc rotation and the location of VF defects was evaluated with Kappa (κ) value. [19] A κ value between 0.81 and 1 indicates almost perfect agreement, between 0.61 and 0.80 indicates substantial agreement, between 0.41 and 0.60 indicates moderate agreement, between 0.21 and 0.40 indicates fair agreement, and between 0 and 0.2 indicates slight agreement.

Inter-observer reproducibility of the measurement of tilt ratio, optic disc rotation degree, and β-zone PPA-to-disc area ratio was assessed by calculation of the intraclass correlation coefficients (ICCs) with 95% confidence intervals (CIs) in all eyes. Clinical data were collected on a spreadsheet in Excel for Mac 2012 version 10.11.5 (MacBook Air, Apple, Shenzhen, Guangdong, China) and analyzed using SPSS version 18.0 (SPSS Inc., Chicago, IL, USA). A *P* value of less than 0.05 was considered statistically significant.

## Results

The study included 100 eyes of 50 myopic subjects (mean age, 50.1 ± 10.0 years; range, 27–70 years; 30 men (60.0%); all Taiwanese) with unilateral glaucomatous VF defects. Individual participants’ data are presented in S1 Table. Twenty-six subjects (52%) had VF defects in the right eyes and 24 subjects (48%) had VF defects in the left eyes. The ICCs for the measurement of the tilt ratio, optic disc rotation degree and β-zone PPA-to-disc area ratio were 0.973 (95% CI, 0.960–0.982), 0.967 (95% CI, 0.952–0.978) and 0.995 (95% CI, 0.993–0.997), respectively.

Table 1 revealed the demographic data of the eyes with VF defects and the contralateral eyes without VF defects. The eyes with VF defects had significantly larger MDs and LVs (4.60 ± 3.48 dB and 49.19 ± 31.69 dB, respectively) than the contralateral eyes (-1.12 ± 1.14 dB and 2.37 ± 1.36 dB, respectively; both *P* < 0.001). Eyes with VF defects had significantly more myopia and longer AL (*P* = 0.001 and 0.013, respectively). Both IOP and CCT were not significantly different between the two groups. With regard to the optic disc morphology, the tilt ratio did not reach the significant difference between the eyes with VF defects (1.36 ± 0.17) and the contralateral eyes (1.34 ± 0.19, *P* = 0.437). The degree of optic disc rotation was significantly greater in the eyes with VF defects (12.96 ± 7.21°) than the contralateral eyes (6.86 ± 4.30°, *P* < 0.001). The β-zone PPA-to-disc area ratio was also significantly greater in the eyes with VF defects (0.99 ± 0.51) than the contralateral eyes (0.79 ± 0.65, *P* = 0.024).

**Table 1:** Demographic data of the eyes with visual filed (VF) defects and the contralateral eyes without VF defects in 50 subjects with primary open-angle glaucoma.

Parameters	Eyes with VF defects	Contralateral eyes without VF defects	*P* value*
**Mean defect (dB)**	4.60 ± 3.48	-1.12 ± 1.14	<0.001^+^
**Loss variance (dB)**	49.19 ± 31.69	2.37 ± 1.36	<0.001^+^
**Spherical equivalence (D)**	-6.22 ± 3.28	-5.65 ± 3.28	0.001^+^
**Axial length (mm)**	26.41 ± 1.32	26.24 ± 1.37	0.013^+^
**Intraocular pressure (mmHg)**	14.92 ± 2.70	14.80 ± 2.71	0.589
**Central corneal thickness (μm)**	549.82 ± 39.72	550.32 ± 39.99	0.710
**Optic disc tilt ratio**	1.36 ± 0.17	1.34 ± 0.19	0.437
**Optic disc rotation degree**	12.96 ± 7.21	6.86 ± 4.30	<0.001^+^
**β-zone PPA-to-disc area ratio**	0.99 ± 0.51	0.79 ± 0.65	0.024^+^

D = diopter; dB = decibel; PPA = parapapillary atrophy.

Data are expressed as mean ± standard deviation.

* Paired *t* test.

^+^ *P* < 0.05.

Table 2 showed the comparison of optic disc parameters measured using SD-OCT. The disc area was similar in the eyes with VF defects and the companions (*P* = 0.954). The rim area and average RNFL thickness in the eyes with VF defects were significantly less than those in the contralateral eyes (both *P* < 0.001). The cup volume and vertical cup-to-disc ratio in the eyes with VF defects were significantly larger than those in the contralateral eyes (both *P* < 0.001).

**Table 2:** Comparison of optic disc parameters in spectral-domain optical coherence tomography between the eyes with visual filed (VF) defects and the contralateral eyes without VF defects in 50 subjects with primary open-angle glaucoma.

Parameters	Eyes with VF defects	Contralateral eyes without VF defects	*P* value*
**Disc Area (mm**^**2**^**)**	1.81 ± 0.40	1.81 ± 0.44	0.954
**Rim Area (mm**^**2**^**)**	0.78 ± 0.20	0.97 ± 0.22	<0.001^+^
**Cup volume (mm**^**3**^**)**	0.39 ± 0.23	0.30 ± 0.21	<0.001^+^
**Vertical C/D ratio**	0.75 ± 0.10	0.65 ± 0.12	<0.001^+^
**Average RNFL thickness (μm)**	73.80 ± 11.60	85.48 ± 10.35	<0.001^+^

C/D ratio = cup-to-disc ratio; RNFL = retinal nerve fiber layer.

Data are expressed as mean ± standard deviation.

* Paired *t* test.

^+^ *P* < 0.05.

In the univariate logistic regression analysis, the degree of optic disc rotation and average RNFL thickness were significantly associated with the presence of VF defects (both *P* < 0.001). In the multivariate analysis, the degree of optic disc rotation and average RNFL were still significantly associated with the presence of VF defects after adjustment for SE, AL, IOP, CCT, tilt ratio, β-zone PPA-to-disc area ratio, and disc area (both *P* < 0.001) (Table 3).

**Table 3:** Logistic regression analysis with the dependent variable being the presence of visual field defects.

Adjusted Factors	Univariate Analysis			Multivariate Analysis		
	Odds Ratio	95% Confidence Interval	*P* Value	Odds Ratio	95% Confidence Interval	*P* Value
**Spherical equivalence (D)**	0.948	0.839–1.070	0.386	1.015	0.748–1.377	0.923
**Axial length (mm)**	1.103	0.821–1.481	0.517	0.947	0.482–1.859	0.874
**Intraocular pressure (mmHg)**	1.016	0.878–1.176	0.829	0.891	0.711–1.115	0.313
**Central corneal thickness (μm)**	1.000	0.990–1.010	0.949	0.998	0.983–1.013	0.789
**Optic disc tilt ratio**	1.808	0.199–16.409	0.599	2.799	0.044–179.890	0.628
**Optic disc rotation degree**	1.219	1.109–1.340	<0.001^+^	1.310	1.143–1.503	<0.001^+^
**β-zone PPA-to-disc area ratio**	1.882	0.888–3.989	0.099	1.184	0.318–4.405	0.802
**Disc area (mm**^**2**^**) on SD-OCT**	1.020	0.396–2.624	0.967	0.647	0.185–2.259	0.495
**Average RNFL thickness (μm) on SD-OCT**	0.906	0.865–0.948	<0.001^+^	0.888	0.841–0.938	<0.001^+^

D = diopter; PPA = parapapillary atrophy; RNFL = retinal nerve fiber layer; SD-OCT = spectral-domain optical coherence tomography.

^+^ *P* < 0.05.

**Fig 2:**
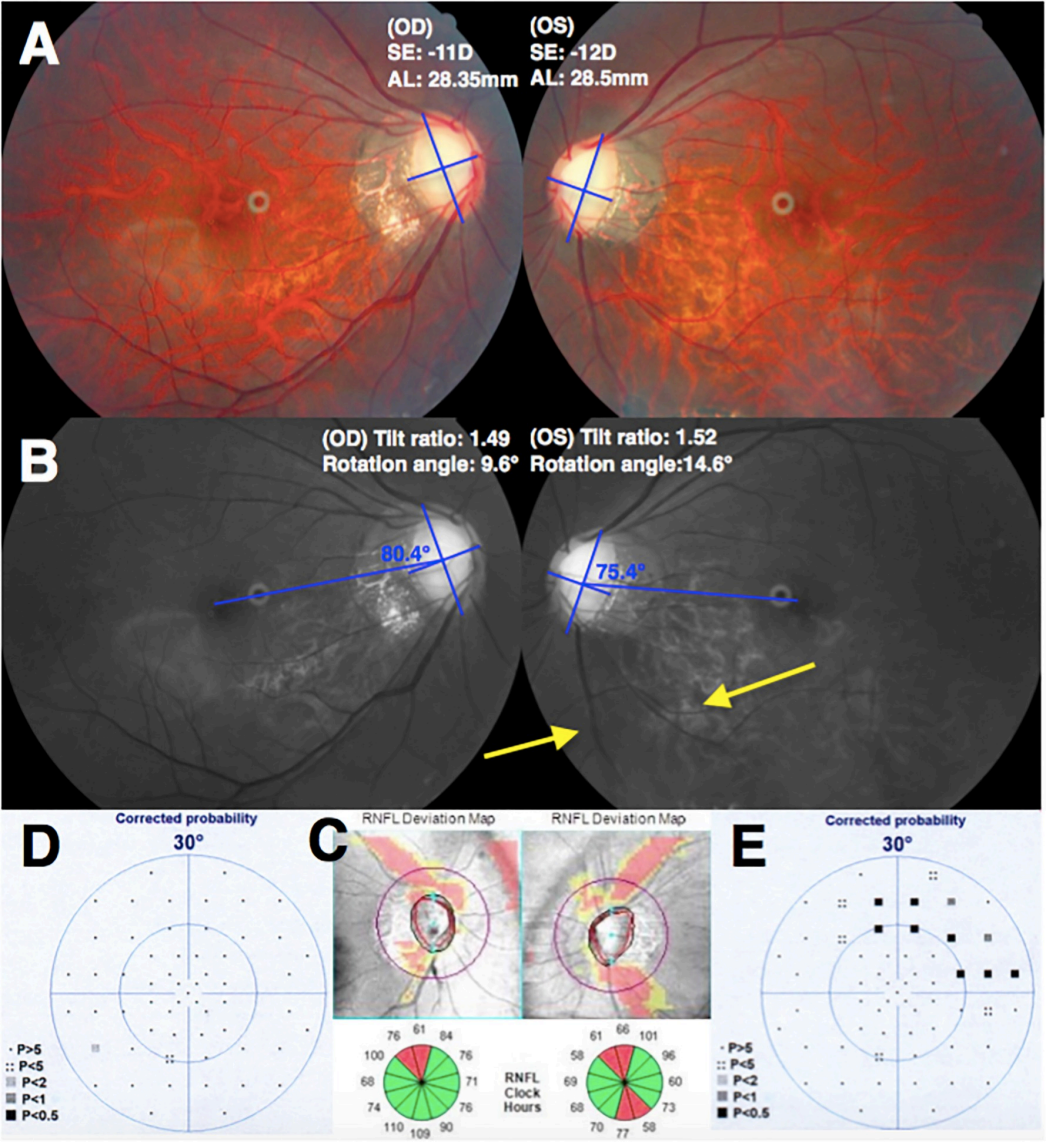
The more myopic eye had greater optic disc rotation and glaucomatous visual field (VF) defect. (A) Fundus photographs of a 27-year-old woman with primary open-angle glaucoma and myopia (spherical equivalent [SE], -11 diopters [D]; axial length [AL], 28.35 mm in the right eye and SE, -12 D; AL, 28.5 mm in the left eye). (B) The optic disc of right eye showed a tilt ratio of 1.49 and inferior rotation degree of 9.6°. The optic disc of left eye showed a tilt ratio of 1.52 and inferior rotation degree of 14.6°. Inferotemporal retinal nerve fiber layer (RNFL) defect in the left eye was found (arrow). (C) In the RNFL deviation map of spectral-domain optical coherence tomography (SD-OCT) imaging, there was inferotemporal RNFL defect in the left eye. (D, E) Superior arcuate VF defect in the left eye was found. The mean defect on automated perimetry was 0.9 decibels (dB) in the right eye and 4.1 dB in the left eye.

Table 4 showed the demographic data of the subgroup with the more myopic eyes accompanied with VF defects and the contralateral less myopic eyes without VF defects. The IOP and CCT were similar between the paired eyes. The tilt ratio was not significantly different between the paired eyes (*P* = 0.549). The degree of optic disc rotation was significantly greater in the more myopic eyes with VF defects (12.85 ± 7.36°) than the contralateral less myopic eyes without VF defects (6.93 ± 4.46°, *P* < 0.001). β-zone PPA-to-disc area ratio was significantly greater in the more myopic eyes with VF defects (1.05 ± 0.53) than the contralateral less myopic eyes without VF defects (0.81 ± 0.65, *P* = 0.020). Fig 2 showed a representative case of the more myopic eye had greater optic disc rotation accompanied with glaucomatous VF defect.

**Table 4:** Demographic data of the more myopic eyes with visual field (VF) defects and the contralateral less myopic eyes without VF defects in 38 subjects with primary open-angle glaucoma.

Parameters	More myopic eyes with VF defects	Contralateral less myopic eyes without VFdefects	*P* value*
**Mean defects (dB)**	4.54 ± 2.83	-1.00 ± 1.18	<0.001^+^
**Loss variance (dB)**	49.10 ± 31.83	2.41 ± 1.39	<0.001^+^
**Spherical equivalence (D)**	-6.47 ± 3.49	-5.39 ± 3.39	<0.001^+^
**Axial length (mm)**	26.38 ± 1.36	26.03 ± 1.37	<0.001^+^
**Intraocular pressure (mmHg)**	14.90 ± 2.73	14.70 ± 2.58	0.391
**Central corneal thickness (μm)**	553.29 ± 33.80	554.03 ± 36.74	0.580
**Disc Area (mm**^**2**^**)**	1.82 ± 0.42	1.86 ± 0.47	0.624
**Optic disc tilt ratio**	1.36 ± 0.18	1.34 ± 0.20	0.549
**Optic disc rotation degree**	12.85 ± 7.36	6.93 ± 4.46	<0.001^+^
**β-Zone PPA-to-disc area ratio**	1.05 ± 0.53	0.81 ± 0.65	0.020^+^

D = diopter; dB = decibel; PPA = parapapillary atrophy.

Data are expressed as mean ± standard deviation.

* Paired *t* test.

^+^ *P* < 0.05.

**Fig 3:**
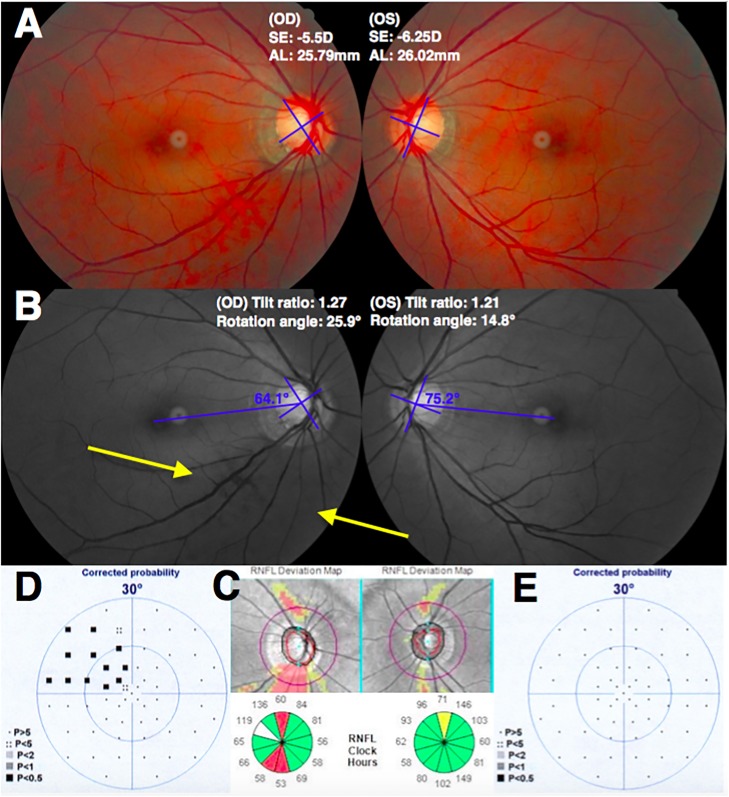
The less myopic eye had greater optic disc rotation and glaucomatous visual field (VF) defect. (A) Fundus photographs of a 49-year-old woman with primary open-angle glaucoma and myopia (spherical equivalent [SE], -5.5 diopters [D]; axial length [AL], 25.79 mm in the right eye and SE, -6.25 D; AL, 26.02 mm in the left eye). (B) The optic disc of right eye showed a tilt ratio of 1.27 and inferior rotation degree of 25.9°. The optic disc of left eye showed a tilt ratio of 1.21 and inferior rotation degree of 14.8°. Inferotemporal retinal nerve fiber layer (RNFL) defect in the right eye was found (arrow). (C) In the RNFL deviation map of spectral-domain optical coherence tomography (SD-OCT) imaging, there was inferotemporal RNFL defect in the right eye. (D, E) Superior nasal step VF defect in the right eye was found. The mean defect on automated perimetry was 3.0 decibels (dB) in the right eye and -1.5 dB in the left eye.

Table 5 showed the demographic data of the subgroup of the more myopic eyes without VF defects and the contralateral less myopic eyes accompanied with VF defects. The IOP and CCT were similar between the paired eyes. The tilt ratio and β-zone PPA-to-disc area ratio were not significantly different between the paired eyes. Only the degree of optic disc rotation was significantly greater in the less myopic eyes with VF defects (13.31 ± 7.00°) than the contralateral more myopic eyes without VF defects (6.64 ± 3.91°, *P* = 0.005). Fig 3 showed a representative case of the less myopic eye had greater optic disc rotation accompanied with glaucomatous VF defect.

**Table 5:** Demographic data of the less myopic eyes with visual field (VF) defects and the contralateral more myopic eyes without VF defects in 12 subjects with primary open-angle glaucoma.

Parameters	Less myopic eyes with VF defects	Contralateral more myopic eyes without VF defects	*P* value*
**Mean defect (dB)**	4.81 ± 5.18	-1.50 ± 0.93	0.001^+^
**Loss variance (dB)**	49.46 ± 32.64	2.24 ± 1.31	<0.001^+^
**Spherical equivalence (D)**	-5.41 ± 2.46	-6.49 ± 2.88	0.001^+^
**Axial length (mm)**	26.50 ± 1.23	26.91 ± 1.20	0.004^+^
**Intraocular pressure (mmHg)**	14.99 ± 2.75	15.14 ± 3.21	0.774
**Central corneal thickness (μm)**	538.83 ± 54.87	538.58 ± 48.85	0.949
**Disc Area (mm**^**2**^**)**	1.77 ± 0.32	1.64 ± 0.26	0.150
**Optic disc tilt ratio**	1.34 ± 0.15	1.32 ± 0.18	0.598
**Optic disc rotation degree**	13.31 ± 7.00	6.64 ± 3.91	0.005^+^
**β-zone PPA-to-disc area ratio**	0.80 ± 0.37	0.75 ± 0.66	0.771

D = diopter; dB = decibel; PPA = parapapillary atrophy.

Data are expressed as mean ± standard deviation.

* Paired *t* test.

^**+**^ *P* < 0.05.

Among the 33 eyes (66%) with hemifield VF defects, 22 eyes (66.7%) had superior VF defects and 11 eyes (33.3%) had inferior VF defects. The similar percentage of superior VF defects (69.9%) was reported by Park *et al*. [18] The percentage (75.8%) of eyes with the region of RNFL damage being consistent with the direction of optic disc rotation in this study was higher than that (60.9%) reported by Sung *et al*. [20] The κ value for the measures of agreement between the direction of optic disc rotation and the location of VF defects was 0.478 (*P* = 0.006). There was moderate agreement between the direction of optic disc rotation and the location of VF defects.

## Discussion

In clinical practice, we observed that more glaucomatous VF defects may be presented in less myopic eyes, rather than more myopic eyes. There might be factors other than refractive error or AL that are associated with glaucomatous VF defects in myopic eyes. The elongation of AL caused by myopia may result in higher scleral tension across the lamina cribrosa and greater deformation of the ONH than the emmetropic eyes. [28] The deformation of ONH might contribute to a more vulnerable ONH which may associate with higher susceptibility to glaucoma in myopic eyes. The refractive error or AL was not associated with more glaucomatous VF defects in present study. We found optic disc rotation was the most significant structural change among the myopic ONH deformation in predicting the presence of VF defects in the myopic subjects with POAG.

In this study, we found the degree of optic disc rotation in eyes with VF defects was statistically greater than that in the contralateral eyes without VF defects (*P* < 0.001). In both univariate and multivariate logistic regression analyses, the degree of optic disc rotation was significantly associated with the presence of VF defects (both *P* < 0.001). However, the refractive error and AL were not significantly associated with the presence of VF defects. The more degree of optic disc rotation might indicate more ONH deformation which could be associated with higher susceptibility of glaucomatous damage.

In Korea, Lee *et al* reported similar results that the degree of optic disc rotation in glaucomatous VF-affected eyes was larger than the contralateral eyes without VF defect in young myopic subjects. [19] The subjects were younger (mean age, 36.0 ± 6.52 years) and less myopic (mean AL < 26mm) compared to our study. Additionally, they also found AL was not associated with the presence of glaucomatous VF defect in the myopic subjects. On the other hand, Sawada *et al* and Lee *et al* did not find significant correlation between the degree of optic disc rotation and VF defects in myopic subjects with open-angle glaucoma. [26,29] The inconsistency might be explained by the different study designs, study populations, severity of glaucoma, and degree of myopia.

In present study, we particularly showed the subgroup that the degree of optic disc rotation in less myopic eyes accompanied with VF defects was statistically greater than in the contralateral more myopic eyes without VF defects (*P* = 0.005). Therefore, the degree of optic disc rotation should be more crucial than refractive error or AL in predicting the presence of glaucomatous VF defects in myopic subjects. The normal arcuate nerve fibers occupy the superior and inferior temporal portions of the ONH and arch above and below the fovea of fibers temporal to the ONH. The optic disc rotation was defined as the deviation of the longest diameter of disc from the vertical meridian, which was a vertical line perpendicular to a reference line connecting the fovea and the center of disc. The reference line of optic disc rotation measurement may equal to the location of fibers temporal to the ONH which separate arcuate nerve fibers into superior and inferior portions. Thus the presence of optic disc rotation represents the nerve fibers deviate from the normal position and greater degree of optic disc rotation might imply greater nerve fibers deviation and strain which could make nerve fibers more vulnerable to glaucomatous damage.

Park *et al* revealed the direction of optic disc rotation may predict the location of VF damage in Korean with normal tension glaucoma (NTG). [18] Lee *et al* demonstrated the fair agreement (κ = 0.375, *P* = 0.015) between the direction of optic disc rotation and the location of VF defects in Korean myopic patients with NTG. [19] In present study, there was moderate agreement (κ = 0.478, *P* = 0.006) between the direction of optic disc rotation and the location of VF defects. The fair to moderate agreement between the direction of optic disc rotation and the location of VF defects in the two studies might implicate that the direction of optic disc rotation cannot well predict the region of RNFL damage. Due to the influence of optic disc rotation on nerve axons and the vulnerability of superior or inferior nerve axons may be different among individuals, the direction of optic disc rotation cannot always predict the region of axonal damage.

In this study, we did not find significant correlation between the optic disc tilt and glaucomatous VF defects. The same finding was noted by Lee *et al*. [19] In Taiwan, Chen *et al* reported that the greater optic disc tilt was associated with more severe glaucomatous optic neuropathy in highly myopic eyes with POAG. [15] Both Sawada *et al* and Lee *et al* found that greater optic disc tilt ratio was significantly associated with more glaucomatous VF defects by intra-individual comparison in myopic subjects with open-angle glaucoma. [26,29]

The conflicting results might be explained by all studies used the tilt ratio to quantify the amount of the deformation of ONH. However, the two-dimensional analysis by tilt ratio might not actually reflect the real three-dimensional structure of the ONH. The other reason may be that the percentages of subjects with parafoveal VF defects were different between studies. VF defects in early glaucoma are usually detected peripherally and the parafoveal scotomas associated with papillomacular bundle usually develop during the end stage of glaucoma. However in myopic eyes with POAG, the papillomacular bundle may be more vulnerable in the early stage of glaucoma. Kimura *et al* demonstrated that high myopia and tilted disc were significantly associated with papillomacular bundle defects and parafoveal scotomas in early glaucoma. [16] Sung *et al* also reported that the greater disc tilt was associated with development of parafoveal scotoma in myopic eyes with early NTG. [17] During myopia progression, eyeball elongation resulted in scleral stretching and tilting of the optic disc. Myopic tilting of the optic disc might strain the temporal side of lamina cribrosa and axons of papillomacular bundle which make papillomacular bundle more vulnerable to glaucomatous damage. Since the optic disc tilt might be associated with parafoveal scotomas, the different percentages of subjects with parafoveal VF defects would lead to the conflicting results.

In present study, the β-zone PPA-to-disc area ratio was not significantly associated with the presence of VF defect in the univariate and multivariate logistic regression analysis. In the subgroup with the more myopic eyes accompanied with VF defects, the β-zone PPA-to-disc area ratio was significantly greater in the more myopic eyes with VF defects than the contralateral eyes. Whereas in the subgroup of the more myopic eyes without VF defects, the β-zone PPA-to-disc area ratio were not significantly different between the paired eyes.

Lee *et al* also revealed that β-zone PPA was not associated with glaucoma in myopic Korean eyes. [19] Nevertheless, both Sawada *et al* and Lee *et al* demonstrated that the β-zone PPA areas were larger in eyes with more VF defects through intereye comparisons. [26,29] The possible explanation is that the clinically visible β-zone PPA might include β-zone PPA and myopic crescent. As both glaucoma and myopia are associated with clinically visible β-zone PPA, the relationship between β-zone PPA and glaucoma becomes more complicated in myopic eyes. [24,25,30] In the more myopic eyes without VF defects, the clinically visible β-zone PPA might be similar to the less myopic eyes accompanied with VF defects.

There are some limitations in this study. Firstly, is its cross-sectional design, a long-term follow-up study is required to clarify whether optic disc rotation is associated with glaucoma progression. Secondly, the IOPs were not measured by Goldmann applanation tonometry which is currently gold standard for IOP measurement. Thirdly, sample size collected in this study is relatively small in a single hospital. Fourthly, as the ONH morphology was measured on two-dimensional fundus photographs, it might not have correctly reflected the true three-dimensional structure of ONH. Further studies may need to use OCT to investigate the correlation between ONH deformation and glaucomatous VF defects in myopic eyes by three-dimensional assessment of the ONH.

In conclusion, among the ONH deformation in myopic eyes, including optic disc tilt, optic disc rotation, and β-zone PPA, only greater degree of optic disc rotation was associated with the presence of VF defects in myopic subjects with POAG. In Taiwan, when diagnosing glaucoma in myopic subjects, we suggest comparing the fundus photographs of both eyes simultaneously to find out the difference in the degree of optic disc rotation between the two eyes as the eye with greater degree of optic disc rotation might be associated with glaucomatous VF defects. Deformation of ONH is a more important factor than refractive error or AL in predicting the presence of VF defects in the myopic eyes with POAG.

## Supporting information

S1 Table Baseline demographics of the whole participants. (XLS)
